# Efficacy of amodiaquine, sulphadoxine-pyrimethamine and their combination for the treatment of uncomplicated *Plasmodium falciparum *malaria in children in Cameroon at the time of policy change to artemisinin-based combination therapy

**DOI:** 10.1186/1475-2875-9-34

**Published:** 2010-01-27

**Authors:** Wilfred F Mbacham, Marie-Solange B Evehe, Palmer M Netongo, Isabel A Ateh, Patrice N Mimche, Anthony Ajua, Akindeh M Nji, Domkam Irenee, Justin B Echouffo-Tcheugui, Bantar Tawe, Rachel Hallett, Cally Roper, Geoffrey Targett, Brian Greenwood

**Affiliations:** 1 Biotechnology Centre, University of Yaoundé I, Box 8094 Yaoundé, Cameroon; 2 Department of Infectious and Tropical Diseases, London School of Hygiene and Tropical Medicine, London, UK

## Abstract

**Background:**

The efficacy of amodiaquine (AQ), sulphadoxine-pyrimethamine (SP) and the combination of SP+AQ in the treatment of Cameroonian children with clinical malaria was investigated. The prevalence of molecular markers for resistance to these drugs was studied to set the baseline for surveillance of their evolution with time.

**Methods:**

Seven hundred and sixty children aged 6-59 months with uncomplicated falciparum malaria were studied in three ecologically different regions of Cameroon - Mutengene (littoral equatorial forest), Yaoundé (forest-savannah mosaic) and Garoua (guinea-savannah). Study children were randomized to receive either AQ, SP or the combination AQ+SP. Clinical outcome was classified according to WHO criteria, as either early treatment failure (ETF), late clinical failure (LCF), late parasitological failure (LPF) or adequate clinical and parasitological response (ACPR). The occurrence of mutations in *pfcrt, pfmdr1, dhfr *and *dhps *genes was studied by either RFLP or dot blot techniques and the prevalence of these mutations related to parasitological and therapeutic failures.

**Results:**

After correction for the occurrence of re-infection by PCR, ACPRs on day 28 for AQ, SP and AQ+SP were 71.2%, 70.1% and 80.9%, in Garoua, 79.2%, 62.5%, and 81.9% in Mutengene, and 80.3%, 67.5% and 76.2% in Yaoundé respectively. High levels of *Pfcrt *76T (87.11%) and *Pfmdr1 *86Y mutations (73.83%) were associated with quinoline resistance in the south compared to the north, 31.67% (76T) and 22.08% (86Y). There was a significant variation (p < 0.001) of the prevalence of the SGK haplotype between Garoua in the north (8.33%), Yaoundé (36.29%) in the savannah-forest mosaic and Mutengene (66.41%) in the South of Cameroon and a weak relation between SGK haplotype and SP failure. The 540E mutation on the *dhps *gene was extremely rare (0.3%) and occurred only in Mutengene while the *pfmdr1 *1034K and 1040D mutations were not detected in any of the three sites.

**Conclusion:**

In this study the prevalence of molecular markers for quinoline and anti-folate resistances showed high levels and differed between the south and north of Cameroon. AQ, SP and AQ+SP treatments were well tolerated but with low levels of efficacy that suggested alternative treatments were needed in Cameroon since 2005.

## Background

The primary tool for the control of malaria in many parts of Africa remains the early diagnosis and treatment of clinical cases of malaria, a policy that is threatened by increasing resistance of *Plasmodium falciparum *to many of the cheap and previously effective anti-malarial drugs, such chloroquine (CQ), amodiaquine (AQ) and sulphadoxine-pyrimethamine (SP).

By the beginning of the twenty-first century, most malaria infections in Cameroon were reported to be resistant to chloroquine [1-4]. Thus, in 2002, the Cameroonian Ministry of Health recommended a change to the use of AQ and SP as first- and second-line treatment for uncomplicated malaria, respectively, with SP being used for intermittent preventive treatment in pregnancy. In two previous studies conducted in 1998/2000 in children between five and 14 years of age in the extreme south of Cameroon [4], clinical and parasitological failure rates for SP, AQ and the AQ+SP combination of 13.6%, 10.2% and 0% respectively were reported. In 2003, clinical and parasitological responses to treatment with SP of 46.6% and 43.5% respectively were reported in Nkambe and Mutengene in the west and southwest of Cameroon respectively [5]. As a result, there was an increased use of AQ alone or combined with SP in many areas, exposing the *P. falciparum *population to sub-optimal drug concentrations with the potential for the emergence of drug resistance. Resistance of *P. falciparum *to SP is due to point mutations in the dihydrofolate reductase *(dhfr) *and dihydropteroate synthase *(dhps) *genes [6]. The molecular mechanism of AQ resistance in P. *falciparum *remains uncertain but may be associated with mutations in the *pfcrt76T *and *pfmdr1 86Y *genes [7,8]. To evaluate the performance of SP, AQ, and the combination in Cameroon and to set a baseline for monitoring the evolution of resistance, a three-arm, double-blind randomized, efficacy and safety study of AQ, SP, and AQ+SP for the treatment of uncomplicated *P. falciparum *malaria was conducted in young Cameroonian children in 2004-2006, with molecular characterization of parasites obtained from patients who failed treatment with these drugs.

## Methods

### Study design

**Figure 1:**
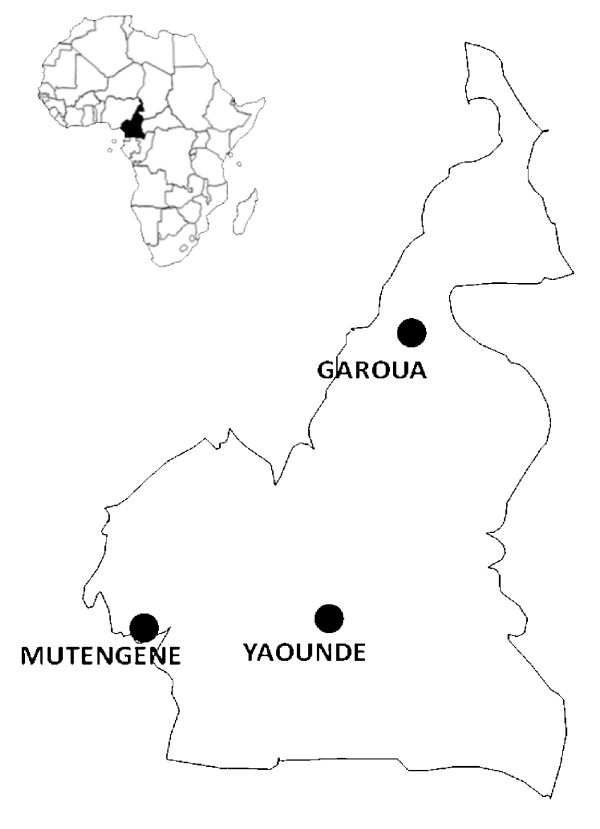
**Study Sites for AQ, SP and SPAQ Efficacy in Cameroon**. Mutengene (forest-littoral), Yaoundé (forest-savannah mosaic), and Garoua (guinea-savannah).

A double-blind, randomized, controlled trial of AQ, SP and the combination AQ+SP, in the treatment of children with uncomplicated falciparum malaria was carried out in three different ecological zones of Cameroon - Mutengene (forest-littoral), Yaoundé (forest-savannah mosaic), and Garoua (guinea-savannah) (Figure 1). Malaria transmission at each of the three sites has two peaks periods, at the start and at the end of the rainy season which lasts from April to October in Mutengene midway peri-urban town between Limbe and Tiko (EIR = 160-287) [9] and Yaoundé urban/rural (EIR = 34-172)[10] and from May to September in Garoua (close to a rice farming field), similar to Goulmoun in Chad (EIR= 311) [11]. Outpatient clinics of the Cameroon Baptist Convention, Health Board clinics and the Hôpital Jesus, Sauve et Guérit participated in recruitment at Mutengene, Yaoundé and Garoua, respectively.

### Participant recruitment

Informed consent for enrolment into the trial was obtained from the parents or guardians of children aged 5-59 months, who presented to one of the study clinics with an axillary temperature between 37.5°C and 40°C or with a history of fever in the past 24 hours and *P. falciparum *parasitaemia of between 1,000 and 200,000 parasites/μl. Children were excluded if they were unable to tolerate oral medication, had any signs indicative of severe or complicated malaria, had a chronic underlying disease or had sensitivity to any of the trial medications [9]. Children were block-randomized in 20s, using computer-generated tables into three groups to receive AQ, SP or AQ+SP. Demographic and clinical information was entered on to the case report form.

### Treatment and follow-up

Drugs were purchased from the National Central Pharmacy through the Cameroon Baptist Convention and were quality controlled by the analytical laboratory in Mutengene, Cameroon. Children in the groups who received monotherapy, received placebos of the complimentary drug purchased from Kakwa BioFarm Accra, Ghana and matched with the active drugs for colour and shape. The first treatment dose was administered at the clinic by the pharmacy attendant and repeated if the child vomited within 30 minutes of administration. The second and third doses of treatment were administered at home by the parent or guardian. Adherence was verified on day 3 by a field worker who asked the parent or guardian about prior drug administration and asked to see any remaining tablets or blisters. Doses were administered according to a dose per weight schedule. All children were given a single dose of paracetamol, 250 mg, 30 min before administration of the trial drug, to control fever. Amodiaquine-SP was administered either as monotherapies or as a combination at 10 mg/kg/day amodiaquine for 3 days and/or half a tablet containing 250 mg sulphadoxine and12.5 mg pyrimethamine for a 10 kg body weight of SP Children were assessed either in the hospital or through home visits on days 3, 7, 14, and 28, and scored as either early treatment failure (ETF), late clinical failure (LCF), late parasitological failure (LPF) or adequate clinical and parasitological response (ACPR) [12]. Rescue medication was quinine sulphate, given in a dose of 30 mg/kg/day over a 7 day period in line with Cameroonian National Treatment guidelines. This drug was administered to children with persistent symptomatic parasitaemia and to those with parasites on day 28 regardless of fever. Filter paper sample were collected for molecular parasitology analysis. There were no serious adverse effects to be reported to a Data and Safety Monitoring Board.

### Laboratory analyses

Field's stain of thick blood films was used to screen children before recruitment when 20 high-power fields were examined. Thick films stained with Giemsa were used for definitive parasites counts; 200 high power fields were screened before a slide was declared negative. The number of asexual forms of *P. falciparum *per 200 leucocytes was recorded and converted into parasite density per μl by assuming an average white cell count of 8,000/μl. Gametocytes were counted against 500 leukocytes. The mean of 2 slide readings was performed and discrepancies greater than 10% were performed by the microscopy quality assurance expert. Haemoglobin (Hb) concentrations were measured with an electronic haemoglobin reader (Hemocue^®^, Sweden). Parasite DNA was extracted from filter paper using the Chelex method as described previously [13]. *Msp1, msp2 *and *glurp *genes were amplified through 35 rounds of PCR using primers obtained from MR4, USA as described [14] to differentiate between recrudescence and re-infections. PCR amplifications of the *dhfr *and *dhps *genes were performed with primers obtained from MR4 as described by Durasingh *et al *[15]. The PCR products were separated by electrophoresis in a 1.5% agarose gel in the presence of ethidium bromide. The 100 bp ladder sold by the New England Biolabs was used as DNA size standard for molecular size determination. The sequence-specific oligonucleotide polymorphism (SSOP) technique was used for molecular genotyping of point mutations of *pfcrt*, *pfmdr-1, dhfr *and *dhps *genes as previously described by Pearce *et al *[16]. Heat denatured (95°C for 2 minutes), nested PCR products encompassing polymorphisms were spotted onto nylon membranes alongside other PCR products of known sequence polymorphism representing all common variants of the relevant gene. Replicate blots were made of each gene set so that simultaneous probing with other oligonucleotide probes was possible. Corresponding sequence-specific oligonucleotide probes that were 3'-end labelled with digoxigenin (DIG) were used for hybridization. SNP-specificity during hybridization was ensured by high stringency with TMAC (Tetramethyl Ammonium Chloride) washes. Alkaline phosphatase-conjugated anti-DIG Fab fragments were used to detect DIG-labelled probes as described by Conway *et al *[17]. Visualization was performed by enhanced chemifluorescence and detected on a phosphoimager (Molecular Dynamics Storm 840; Amersham Pharmacia Biotech). The presence or absence of the sequence polymorphism was scored after adjusting for background. Samples were scored as mixed haplotypes when the value of the intensity of the reaction of a SNP was about half that of the majority positive SNP. Mixed haplotypes of equal strength were discarded in frequency calculations in favour of either a single or majority type. This principle was used both for *dhfr *and *dhps *genes. Indeterminate samples by dot blot of *dhfr *or *dhps *were restriction digested for the RFLP [13,15] and re-scored.

### Statistical analysis

Data were double entered into a database created with Microsoft Access and discordances were resolved before range and consistency checks were performed. An Exploratory Data Analysis (EDA) was done to provide measures of central tendency and dispersion (mean, geometric mean, median, standard deviation, range, 95%CI values). PCR-adjusted parasitological cure rates between the treatment arms were compared using χ^2 ^test. The association between various covariates (age, gender, ethnicity, genetic markers for drug resistance) and therapeutic outcome was performed by a multiple logistic regression model in which progressive selection of significant parameters and the interactions between the different covariates were investigated. These models took into account the different ecological zones where the trial was conducted to examine geographical differences in response rates. The Mantel Haenszel test was used when necessary for stratification, The Kruskal Wallis test was used when continuous variables (such as parasitaemia) where not normally-distributed. MicroSoft Excel 2003 was used for producing graphs.

### Ethics

Ethical approval for the study was obtained from the Institution Review Board of the Cameroon Baptist Convention Health Board, the national ethics committee of the Ministry of Health, Cameroon and from the Ethics Review Committee of the London School of Hygiene and Tropical Medicine. The interests of the patients were guarded by a local safety monitor and by an international Data and Safety Monitoring Board. The trial was registered on the NIH clinical trials database by number-NCT00146718.

## Results

### Patient characteristics

**Figure 2:**
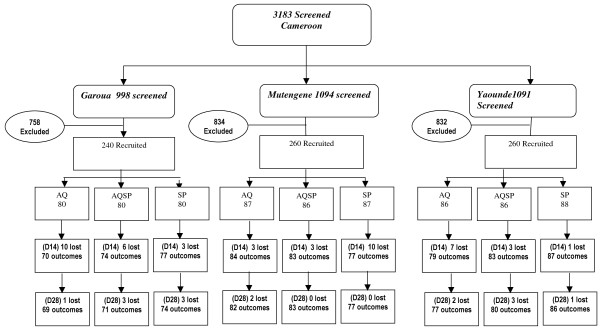
**Number of subjects screened, recruited and followed up by treatment group**.

A total of 3,183 children were screened of whom 760 were eligible for inclusion in the trial. These children were randomized and enrolled across all three sites. About three quarters of the participants were excluded on the basis of severe malaria (17.4%), parasite negativity (37.6%), residence outside the study area (17.1%), fresh scarification marks (3.6%), less than six months of age (19.5%) and refusal of consent (5.8%). Two hundred and fifty five children were allocated to receive SP, 253 to received AQ and 252 to receive SP+AQ (Figure 2). The mean age at enrollment (27-30 months) was similar at each site and between study groups (Table 1). The mean weight per group varied between 10.7 kg and 13.0 kg, with children in Garoua being on average lighter than those from the other sites. Mean parasite density was higher in Mutengene (the forest-littoral zone) than at the other two sites. The prevalence of fever at enrolment was 99%, 97% and 77% in Mutengene, Yaoundé and Garoua respectively. Weakness, loss of appetite and vomiting were the other most frequently recorded symptoms at each site during screening.

**Table 1:** Baseline characteristics at enrollment of study children by study site

Site	Mut			Yao			Gar		
**Drug Groups**	**SPAQ**	**SP**	**AQ**	**SPAQ**	**SP**	**AQ**	**SPAQ**	**SP**	**AQ**

Age (mean in months ± SD)	29 ± 15.5	27 ± 14.8	27 ± 15.2	29.4 ± 14.5	30 ± 15.1	29 ± 13.8	27 ± 15.4	26.6 ± 16.5	27.4 ± 17.2

Female/Male ratio	1	0.9	1	0.7	1.2	0.82	0.74	0.82	1.2

Weight (kg) (mean ± SD)	12.5 ± 3.5	12.2 ± 3.2	12.2 ± 4.1	13.02 ± 3.0	12.6 ± 3.4	12.9 ± 3.2	10.84 ± 3.3	10.86 ± 2.7	10.59 ± 3.3

Geo mean parasitemia (± SD)	39601 (± 48160)	27014 (± 49560)	22072 (± 30260)	7776 (± 27120)	6721 (± 25200)	5364 (± 25720)	7264 (± 27730)	5668 (± 19040)	6294 (± 26890)

Temp °C (mean ± SD)	38.9 ± 1.1	38.7 ± 1.1	38.7 ± 1.0	38.2 ± 1.2	38.3 ± 1.0	38.4 ± 1.2	37.9 ± 0.6	38.0 ± 0.6	37.7 ± 0.7

A, B, C, were groups to which independently at each site drugs were randomly assigned. SP- Sulphadoxine-pyrimethamine, AQ - Amodiaquine, SPAQ- combination of AQ and SP, SD- Standard Deviation. Ecotypes; Mutengene (Mut) is in the littoral-forest, Yaoundé (Yao) is in the forest-savannah mosaic and Garoua (Gar) is in the guinnea-savannah

### Safety, clinical and parasitological outcomes

Follow-up rates were high with 93.1%, 93.5% and 89.2% of children completing the 28 days of follow up in Mutengene, Yaoundé and Garoua respectively (Figure 2). No serious adverse events were reported with the exception of one case of pneumonia in a child who received AQ. Adverse events (Table 2) were considered to be mild to moderate and similar to malaria symptoms. All resolved without sequelae within the 28-day follow-up period. After correction for re-infection by PCR, (ACPRs) on day 14 for AQ, SP and AQ+SP respectively, were 72.0%, 71.0%, and 81.7% in Garoua, 88.3%, 75.3% and 82.5% in Mutengene and 82.3%, 86.3% and 88.0%, in Yaoundé (Table 3). By D28, the ACPRs for AQ, SP and AQ+SP respectively were 71.2%, 70.1% and 80.9% in Garoua, 79.2%, 62.5% and 81.9% in Mutengene and 80.3%, 67.5% and 76.2% in Yaoundé, respectively (Table 3). The efficacy of SP did not differ significantly between sites (p = 0.14). However the efficacy of SP at all sites was lower than that for AQ or SP+AQ (P < 0.05 for all sites combined). Gametocytaemia was detected on day 7 more frequently in children treated with SP than in those treated with AQ or AQ+SP, percentages being 13.9%, 5.0% and 2.4% respectively in Mutengene, 14.5%, 7.1%, and 8.9% in Yaoundé and 2.9%, 0.0% and 1.4% in Garoua (p < 0.05). Haemoglobin concentration rose from a mean of 8.53 ± 1.72 g/dL at enrollment to a mean of 10.56 ± 1.26 g/dL at day 28 across all sites combined with no differences between groups.

**Table 2:** Frequency of common adverse events

Drug	Adverse Events	Frequency
SPAQ	Weakness	1.25%
	Cough	1.25%
	Rashes	0.84%

SP	Weakness	1.25%
	Cough	1.25%
	Rashes	0.84%
	Anorexia	0.84%
	Diarrhea	1.25%

AQ	Weakness	02.5%
	Cough	0.42%
	Rashes	0.42%

Frequencies represented here were calculated across all study sites and represent those whose frequencies exceeded 0.

**Table 3:** Clinical and parasitological outcomes by site and treatment group

		Mutengene			Yaounde			Garoua		
**Treatment**	**group**	**SPAQ**	**SP**	**AQ**	**SPAQ**	**SP**	**AQ**	**SPAQ**	**SP**	**AQ**

ETF		4	14	3	0	8	1	3	10	10

LCF		2	7	4	2	12	4	2	5	1

LPF		6	11	6	10	12	8	6	7	8

ACPR, D14 (PCR corrected)		82.5%	75.3%	88.3%	82.3%	88.0%	86.3%	81.7%	71.0%	72.0%

ACPR, D28 (PCR corrected)		81.9%	62.5%	79.2%	76.2%	67.5%	80.3%	80.9%	70.1%	71.2%

Gametocyte	D3	1	10	3	9	7	4	0	1	1

Carriage(n)	D7	2	11	4	7	14	6	0	2	1
	D14	0	6	0	3	16	0	0	1	0
	D28	0	1	0	0	4	0	0	0	0
	%	2.4%	13.9%	5.0%	8.9%	14.5%	7.1%	0.0%	2.9%	1.4%

A, B, C, were groups to which independently at each site drugs were randomly assigned. SP- Sulphadoxine-pyrimethamine, AQ - Amodiaquine, SPAQ- combination of AQ and SP. ETF - Early Treatment Failure, LCF - Late Clinical Failure, LPF- Late Parastological Failure, ACPR - Adequate Clinical and Parasitological Response.

### Molecular markers of resistance

**Figure 3:**
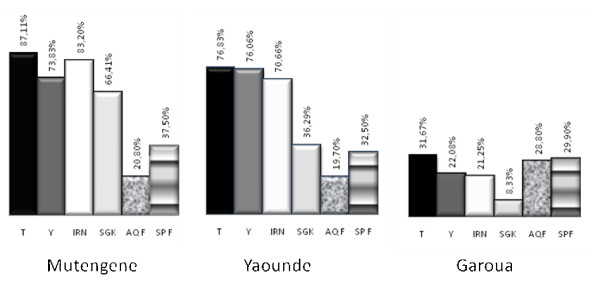
**Prevalence of anti-malarial drug resistance markers in Cameroon**. T represents the threonine (T) mutation on codon 76 (76T) of the *Pfcrt *gene. Y represents tyrosine (Y) mutation at codon 86 (86Y) of the *Pfmdr1 *gene as putative markers for Amodiaquine failure (AQF (D28)). Sulphadoxine-pyrimethamine failure (SPF (D28)) whose molecular markers, IRN represents the isoleucine, arginine and asparagine mutations at codons 51, 59 and 108 (IRN) of the *dhfr *gene and SGK represents the serine, glycine and lysine mutations at codon 436, 437 and 540 of the *dhps *gene:

The prevalence of mutations associated with resistance in the *pfcrt, pfmdr, dhfr *and *dhps *genes in initial blood samples obtained at Mutengene, Yaoundé and Garoua respectively are shown in Figure 3. Although *in vivo *studies suggest no difference in failure rates, between the sites results of molecular markers portray that parasites in the north of the country were less mutant for both the 76T and 86Y respectively of *Pfcrt *and *Pfmdr1 *genes as well as for *dhps *and *dhfr *than parasites in the south. Except for the weak relation between SP failure and the significant variation (p < 0.001) of the prevalence of the SGK haplotype between Garoua in the north (8.33%), Yaoundé (36.29%) in the savannah-forest mosaic and Mutengene (66.41%) in the South of Cameroon (Figure 3), no other associations were found between the prevalence of other resistance mutations at recruitment and subsequent treatment failure at any of the study sites. The 540E *dhps *mutation was detected in 2 cases in Mutengene and nowhere else. The *pfmdr1 *1034K and 1040D mutations were not detected in any of the three sites.

## Discussion

This trial showed that between 2004 and 2006, SP, AQ and AQ+SP were no longer effective enough in the treatment of uncomplicated *falciparum *malaria in Cameroon; SP failing more frequently than AQ or AQ+SP. Cameroon is an area known to harbour parasites with a high level of resistance to chloroquine and this study adds SP and AQ to the list of failing anti-malaria drugs. Although AQ and SP gave satisfactory results up to the late 90's [6], The findings confirm the rapid progression of resistance to SP and AQ between 2001 and 2004 in Cameroon that has been noted by others [18-20]. SP has the potential to further the transmission of resistant parasites by increasing the carriage of gametocytes with subsequent transmission of resistant strains [21,22]. The study demonstrates that the markers of resistance are different between the north and the south of Cameroon. Differences in frequencies of these markers may signify the progression towards full blown resistance rather themselves being considered as indicative of failures. There may be ethnographic or drug pressure differences between the sites that favour higher mutation rates in the south where there is great parasite mixing due to higher transmissions than in the dryer north. Unlike studies in the east of Africa that demonstrated the presence of the 540E mutation on the *dhps *[13,15] this was almost absent (identified only in 2 out of the multitude of samples). This mutation that makes the quintuple mutant so described by others [13,15] for resistance to SP may not be contributing to SP resistance in Cameroon, Central Africa, suggesting that other genes may be involved.

In this study SP, AQ and their combination were generally well tolerated with the main reported side effects being complaints of fatigue in children who received AQ that resolved by D14 and D28. Adverse events were noted if they worsened from D0 or appeared after inclusion. Most of the events described by Olliaro *et al *[23] were only observed with limited frequency post-therapy as indicated under results and may be due to low numbers in this study as well as the fact that the study was conducted in children under the age of 5 who cannot express themselves fully. The administration of the combination of AQ+SP caused no additional adverse events to those usually ascribed to the individual component drugs [23]. AQ and an AQ+SP combination were found to be tolerated. Except for SP, the study treatments were not directly observed and efficacy might have been reduced by lack of compliance with some regimens even though the blister packages were verified on return visits. Similarly, some of the side effects may have been missed during the early stages of their occurrence. Although SP failures seem to be high in the studies related here in, it is still administered to pregnant women as Intermittent Preventive Therapy in pregnancy as part of antenatal care, with the assumption that SP is still efficacious in this adult population with better immunity than in children under 5 years of age. These findings provide useful information for the Ministry of Health, which is recommending amodiaquine-artesunate as first-line treatment of uncomplicated malaria in Cameroon [24], amid fears that it will cause weakness and other side effects. Artesunate+Amodiaquine either as a co-blister or a fixed dose combination (ASAQ), show very high efficacy and tolerability in Cameroon and elsewhere [25-27], negating fears that the ACTs are likely to demonstrate late failures if the component drug (AQ) is already failing.

A high level of resistance mutations was found in the *pfcrt *and *dhfr *genes in keeping with the reduced *in vivo *sensitivity found to AQ and SP. However, a lower prevalence of resistance markers to *pfcrt *and *pfmdr1 *was found in the northern part of the country than in the south but, not matched by significant drop in *in vivo *efficacy. This may be as a result of many factors including greater drug pressure or parasite mixing in the south due to higher transmission intensity than in the dryer north. The genetic structure of parasite in Cameroon may be different from the rest of Africa and further studies are certainly needed especially as the parasites donated to an Africa-wide study of parasite dispersal phenomenon in Africa demonstrated that parasites from Cameroon were one of five types and different form the other four types found in North East, South East, South West and West Africa [16]. These findings suggest that in circumstances of variations across the continent or sub-regions, a different kind of drug policy may be needed, probably embracing multiple first line therapies rather than a one drug-strategy-fits-all approach. Studies of molecular markers can help to map local differences in drug resistance [16]. The continuous mapping of the patterns of resistance mutations to important anti-malarials can be used as an early warning system for therapeutic profiles occurring in endemic areas, leading to discussions on possible changes in drug policy. In conclusion, the high prevalence of *pfcrt, pfmdr1*, *dhfr *and *dhps *mutations and their association with high failure rates *in-vivo to *SP, AQ and the combination SPAQ supports the recommendation by the government of Cameroon in changing its first-line treatment away from monotherapies in favour of artemisinin-based combination therapy and established the baseline for mutations to be monitored over time.

## Competing interests

The authors declare that they have no competing interests.

## Authors' contributions

WFM was in receipt of a Post Doctoral fellowship from the Gates Malaria Partnership at the London School of Hygiene and Tropical Medicine at the time of this study, MSE coordinated sample collection and molecular analysis with IA, PNM, AA, JBE and BT participating in the sample collection and in the conduct of trials in the various sites. PMN performed RFLP and PCR corrections of clinical outcomes and actively participated in the preparation of the manuscript. AN was the data manager and performed the analysis. CR, RH, facilitated the molecular analyses. GT and BG contributed to the study design and critically reviewed the manuscript. All authors read and approved the final manuscript.
